# Comparison of mild and severe forms of hydrocephalus using diffusion tensor imaging in neonatal rats

**DOI:** 10.1186/1743-8454-7-S1-S38

**Published:** 2010-12-15

**Authors:** Weihong Yuan, James P McAllister, Kelley Deren, Francesco Mangano

**Affiliations:** 1 Department of Radiology, Cincinnati Children's Hospital Medical Center, 3333 Burnet Avenue, Cincinnati, Ohio 45229, USA; 2 Department of Neurosurgery, University of Utah, CNC 3230D, Salt Lake City, Utah, 84132, USA; 3 Division of Pediatric Neurosurgery, Cincinnati Children's Hospital Medical Center, 3333 Burnet Avenue, Cincinnati, Ohio 45229, USA

## Background

Abnormal diffusion tensor imaging (DTI) measurements have been found in human and experimental subjects with severe hydrocephalus (HCP) [1-3]. Because ventriculomegaly can often be variable, the present study aimed to investigate the degree of DTI abnormality in neonatal rats with less severe HCP.

## Materials and methods

Seventeen Sprague-Dawley rats were divided into three groups: (1) HCPS - 5 rats with intracisternal kaolin injections leading to severe hydrocephalus; (2) HCPM - 5 rats with the same kaolin injection procedure that developed mild HCP; (3) Control - 7 rats with intracisternal saline injections. Either kaolin or saline injection was performed at P2. DTI were acquired at P9-10 on a 7T Bruker MRI scanner. Evan’s ratios of ventricular size and DTI metrics (Fractional anisotropy (FA) and mean diffusivity (MD)) were calculated and compared using ANOVA and other statistical comparisons.

## Results

The three groups were significantly different (p<0.0001) in Evan’s ratios with HCPM rats (Mean+/-STD = 0.38+/-0.07, Range: 0.31-0.48) slightly higher than controls (Mean+/-STD = 0.29+/-0.01, Range: 0.27-0.31, p=0.02) but lower than the HCPS group (Mean+/-STD=0.85+/-0.04, Range=0.80-0.90, p<0.0001). Both FA and MD values were significantly different among the three groups in cortex, corpus callosum and internal capsule (ANOVA, p<0.05). Post-hoc comparisons showed that the HCPM group was not significantly different from the control group, while the HCPS group was significantly different (lower FA and higher MD, p<0.05) from the control group (with the exception of FA in the internal capsule) and the HCPM group. In the HCPM group, it was found that the MD values in the external capsule increased proportionally with ventricle size (R=0.94, p=0.016), while FA values in the internal capsule decreased with increasing ventricle size (R=-0.98, p=0.005).

## Conclusions

Although the DTI measurements in the HCPM rats with mild ventriculomegaly were not significantly different from normal controls, the correlation between ventricle size and DTI values in this group suggested that different levels of abnormality in brain tissues can be detected with DTI during various stages of hydrocephalus. Our results demonstrate that DTI is potentially a sensitive biomarker for monitoring the progression of ventricle enlargement and may help to determine the optimal window for surgical intervention.

